# Potentiometric Chemical Sensors Based on Metal Halide Doped Chalcogenide Glasses for Sodium Detection

**DOI:** 10.3390/s22249986

**Published:** 2022-12-18

**Authors:** Maria Bokova, Steven Dumortier, Christophe Poupin, Renaud Cousin, Mohammad Kassem, Eugene Bychkov

**Affiliations:** 1 Laboratoire de Phisico-Chimie de l’Atmosphère (LPCA), Université du Littoral Côte d’Opale (ULCO), EA 4493, 59140 Dunkerque, France; 2 Unité de Chimie Environnementale et Interactions sur le Vivant (UCEIV), Université du Littoral Côte d’Opale (ULCO), UR 4492, SFR Condorcet FR CNRS 3417, 59140 Dunkerque, France

**Keywords:** chalcogenide glasses, chemical sensors, mixed cations, conductivity, sodium detection

## Abstract

Chalcogenide glasses are widely used as sensitive membranes in the chemical sensors for heavy metal ions detection. The lack of research work on sodium ion-selective electrodes (Na^+^-ISEs) based on chalcogenide glasses is due to the high hygroscopicity of alkali dopes chalcogenides. However, sodium halide doped Ga_2_S_3_-GeS_2_ glasses are more chemically stable in water and could be used as Na^+^-sensitive membranes for the ISEs. In this work we have studied the physico-chemical properties of mixed cation (AgI)*_x_*(NaI)_30-*x*_(Ga_2_S_3_)_26_(GeS_2_)_44_ chalcogenide glasses (where *x* = 0, 7.5, 15, 22.5 and 30 mol.% AgI) using density, DSC, and conductivity measurements. The mixed cation effect with shallow conductivity and glass transition temperature minimum was found for silver fraction r = Ag/(Na + Ag) ≈ 0.5. Silver addition decreases the moisture resistance of the glasses. Only (AgI)_22.5_(NaI)_7.5_(Ga_2_S_3_)_26_(GeS_2_)_44_ composition was suitable for chemical sensors application, contrary to the single cation sodium halide doped Ga_2_S_3_-GeS_2_ glasses, where 15 mol.% sodium-halide-containing vitreous alloys are stable in water solutions. The analytical parameters of (NaCl)_15_(Ga_2_S_3_)_23_(GeS_2_)_62_; (NaI)_15_(Ga_2_S_3_)_23_(GeS_2_)_62_ and (AgI)_22.5_(NaI)_7.5_(Ga_2_S_3_)_26_(GeS_2_)_44_ glass compositions as active membranes in Na^+^-ISEs were investigated, including detection limit, sensitivity, linearity, ionic selectivity (in the presence of K^+^, Mg^2+^, Ca^2+^, Ba^2+^, and Zn^2+^ interfering cations), reproducibility and optimal pH-range.

## 1. Introduction

The chalcohalide glasses are widely used as sensitive membranes in the Ion Selective Electrodes (ISEs) for potentiometric detection of metal cations and toxic anions. A large number of publications are devoted to the progress with chalcogenide glass-based ISEs in terms of membrane synthesis, fundamental physico-chemical properties, analytical characteristics and the sensing mechanism investigations (see, e.g., the review articles [1,2,3,4,5,6,7,8] and references therein). The chalcogenide glasses have a number of advantages such as the possibility of being synthesized with continuously variable compositions to adjust their physical and chemical properties, and enhanced chemical durability and stability in aggressive media [9]. 

Actually, ISEs with chalcogenide glasses as sensitive membranes for heavy metal ions detection (such as Fe^3+^, Cd^2+^, Pb^2+^, Hg^2+^, Cu^2+^, Ag^+^, Tl^+^ and others [8]) are investigated most extensively, but only one article is devoted to sodium ion-selective electrodes based on chalcogenide glasses [10]. Accurate sodium detection in biological fluids, food and in the environment is extremely important for human health. The main reason of the lack of research on sodium-containing chalcogenide glass membranes is their high hygroscopicity and ability to hydrolyze in aqueous solutions [9]. However, sodium halide doped Ga_2_S_3_-GeS_2_ glasses are more chemically stable in water, and could be used as Na^+^-sensitive membranes for the ISEs [10,11]. Vlasov and Bychkov [10] have studied sodium ISEs based on (NaCl)*_x_*(Ga_2_S_3_)_25−0.25*x*_(GeS_2_)_75−0.75*x*_ vitreous alloys containing 10, 20 and 30 mol.% NaCl and have found that sodium sensors based on (NaCl)_10_(Ga_2_S_3_)_22.5_(GeS_2_)_67.5_ membranes reveal high sensitivity and selectivity towards Na^+^ ions with near-Nernstian response in the concentration range from 10^−3^ to 1 M NaNO_3_ and the detection limit of (1–3) × 10^−4^ M. 

According to ^22^Na tracer and X-ray photoelectron spectroscopy measurements, the sensor sensitivity depends on the direct Na^+^ ion-exchange between the solution and the modified surface layer of the glass membrane [10]. The content of sodium halide and the Ga_2_S_3_/GeS_2_ ratio in the membrane composition could affect the performance of the chemical sensors. Recently, we have investigated the glass-forming regions, macroscopic properties, and Raman spectra in the NaY-Ga_2_S_3_-GeS_2_ (Y = Cl, Br, I) systems [11,12,13,14]. The moisture resistant sodium halide doped Ga_2_S_3_-GeS_2_ glasses could be obtained up to 15 mol.% NaY. According to our preliminary results, the (NaY)_15_(Ga_2_S_3_)_17_(GeS_2_)_68_ glass compositions are promising materials for the Na^+^-ISE membranes with the detection limit of ≈ (6–9) × 10^−5^ M [11]. The most extended glass-forming region in this system can be reached along the (NaY)*_x_*(Ga_2_S_3_)_20+0.2*x*_(GeS_2_)_80−1.2*x*_ composition line up to *x* = 50 mol.% NaY, due to the important role of the chemical interactions between gallium sulfide and metal halide. For this reason, we have decided to investigate analytical characteristics of (NaHal)_15_(Ga_2_S_3_)_23_(GeS_2_)_62_ (Hal = Cl, I) glass membranes in sodium ISEs and to compare them with different NaY-Ga_2_S_3_-GeS_2_ compositions studied previously [10,11].

The addition of silver ions in glass composition could increase its ionic conductivity, which is a very important parameter for the sensors membranes [3]. To our knowledge, the mixed cation Ag^+^/Na^+^ gallium–germanium sulfide vitreous alloys were not studied previously. This system is interesting from both a practical and a theoretical point of view, since only a few research works were related to the mixed cation effect in chalcogenide glasses [15,16,17,18,19,20,21,22,23]. Thus, we intended to synthesize and to characterize the new mixed cation (AgI)*_x_*(NaI)_30-*x*_(Ga_2_S_3_)_26_(GeS_2_)_44_ chalcogenide glasses, where *x* = 0, 7.5, 15, 22.5 and 30 mol.% AgI. The macroscopic characterization of the samples was carried out with the help of the density and differential scanning calorimetry (DSC) analysis. The conductivity measurements were performed using complex impedance spectroscopy. Membrane compositions were chosen according to the results of this detailed physico-chemical analysis. Several key analytical parameters were investigated to evaluate the different glass materials as active membranes in Na^+^-ISEs, including detection limit, sensitivity, linearity, ionic selectivity (in the presence of K^+^, Mg^2+^, Ca^2+^, Ba^2+^ and Zn^2+^ cations, which are the most relevant interfering ions for the sodium determination in biological fluids such as human sweat [24]), reproducibility, and optimal pH-range. The main aim of the work is to study the analytical properties of the fabricated electrodes as a function of the glassy membrane composition.

## 2. Materials and Methods

### 2.1. Glass Synthesis

The typical melt–quenching technique was used for chalcohalide glasses preparation. The compositions studied in this work are (NaHal)_15_(Ga_2_S_3_)_23_(GeS_2_)_62_ (Hal = Cl, I) and (AgI)*_x_*(NaI)_30−*x*_(Ga_2_S_3_)_26_(GeS_2_)_44_ glasses, where *x* = 0, 7.5, 15, 22.5 and 30 mol.% AgI. Commercially available pure gallium, germanium and sulfur elements (5 N), sodium, and/or silver halides (4 N) were mixed according to desired compositions in cleaned silica glass ampoules and sealed under vacuum (10^−6^ mbar). The mixtures were heated at a rate of 1 °C min^−1^ to 950 °C, held at this temperature for a few days with repeated stirring of the melt to ensure homogenization, and quenched in cold water. The tubes (ID/OD of 8/10 mm) with 3 g of melt were quenched horizontally for the macroscopic characterization of the glasses. The (AgI)_30_(Ga_2_S_3_)_26_(GeS_2_)_44_ glass was also prepared with 1 g of melt to facilitate vitrification. The semi-spherical shaped membranes for ISEs were obtained by vertical quenching of the tubes (ID/OD of 10/12 mm) with 1.5 g of the sample. The obtained glasses were annealed for 2 h at 20–30 °C below the glass transition temperature to remove the stress induced during quenching. All synthesized bulk glasses are transparent; their color varies from light yellow to yellow-orange and dark yellow. The photos of the typical glassy samples for the macroscopic characterization and for the ISE membrane are presented in Figure S1a,b (Supplementary material), respectively. 

### 2.2. Glass Characterization

The amorphous nature of materials was confirmed using Bruker D8 Advance diffractometer equipped with a copper anticathode emitting Kα radiation (λ = 1.5406 Å), a LinxEye detector, a goniometer θ/θ and a rotating sample holder. The scattering intensities were measured over the angular range of 10° ≤ 2θ ≤ 80° with a step-size of 0.02° and a count time of 2 s per step. 

The resistance of the glasses to hydrolysis in aqueous solutions was evaluated by keeping them in water for two weeks and controlling the sample mass before and after soaking as well as by visual observation of the glass pieces immersed in water. 

The sample density, *d*, was measured by hydrostatic method with the help of Sartorius YDK 01-0D density kit, using toluene as immersion fluid and a germanium standard (*d*_Ge_ = 5.323 g cm^−3^).

A TA Q200 differential scanning calorimeter (DSC) was used to estimate the glass transition temperature (*T*_g_) of the vitreous alloys. The samples (mass of 10–15 mg) were placed in a standard hermetic aluminum pan and heated with the heating rate of 10 K min^−1^ in the temperature range 25–600 °C under nitrogen flow. The *T*_g_ value was determined as the intersection of two linear portions adjoining the transition elbow of the DSC trace.

The electrical transport of the samples was evaluated by AC conductivity measurements at the temperatures between 20 °C and 200 °C using a Hewlett Packard 4194A impedance meter over the 100 Hz–15 MHz frequency range. The samples were prepared as rectangular plates with a typical area of 6–8 mm^2^ and thickness of 0.7–1.5 mm, polished with SiC powder (9.3 μm grain size) and sputtered with gold on both sides as electrodes. Other details concerning the complex impedance measurements were published elsewhere [12].

### 2.3. Na^+^ Ion Selective Electrodes Fabrication

Three chalcogenide glass compositions were used as sensitive membranes in Na^+^-ISEs sensors: (NaCl)_15_(Ga_2_S_3_)_23_(GeS_2_)_62_; (NaI)_15_(Ga_2_S_3_)_23_(GeS_2_)_62_ and (AgI)_22.5_(NaI)_7.5_(Ga_2_S_3_)_26_(GeS_2_)_44_. For each composition, three electrodes were prepared. The back sides of the semi-spherical as-prepared glasses were polished with SiC powder and then glued into PVC tubes using an electrical isolator epoxy resin and left to dry for few days under an infrared lamp. After this, 2 mL of the NaCl solution (0.1 M) was added inside the PVC tube; this electrolyte ensures the electrical contact between the glass membrane and the AgCl coated silver wire serving as inner reference electrode. Figure S1c (Supplementary material) shows the photo of the fabricated chemical sensor with liquid contact obtained by the end of fabrication process.

### 2.4. Sensors Calibration and Analytical Parameters Measurements

The potentiometric measurements were performed using the following electrochemical cell equation:

Ag,AgCl | KCl_(saturated)_ || KNO_3_ (0.1 M) || NaNO_3_, Ca(NO_3_)_2_, (0.1 M) | glass | NaCl (0.1 M) |Ag, AgCl (1)

Two electrodes were used in the reference (Ag,AgCl | KCl_(saturated)_) electrode and the working glass electrode. The KNO_3_ (0.1 M) was used as a salt bridge. The sensor calibration was performed in magnetically stirred NaNO_3_ solution at 20 °C with the concentration range from 10^−7^ to10^−1^ mol L^−1^ Na^+^ ions. Other details concerning the sensor fabrication and calibration were published elsewhere [25].

The analytical parameters studied in this work are the sensitivity, the detection limit, linearity range, reproducibility, ionic selectivity and optimal pH-range. Selectivity coefficients were determined using the two versions of the mixed solutions methods: (i) under a fixed concentration of the interfering ions (in case of Mg^2+^, Ca^2+^, Ba^2+^ and Zn^2+^ cations) and a variable concentration of the Na^+^ ions or (ii) under a variable concentration of the interfering ions (in case of K^+^) and a constant concentration of the Na^+^ ions. The pH effect on the electrode performance was examined in solutions with constant sodium concentration by adding HCl or NaOH to vary the hydrogen ions concentration. 

## 3. Results

### 3.1. Glass Forming Ability, Moisture Resistance and Physico-Chemical Properties of the AgI-NaI-Ga_2_S_3_-GeS_2_ Glasses

The glass-forming region, macroscopic, and electrical properties for single cation sodium halide doped Ga_2_S_3_-GeS_2_ glasses are already published in [13]. Our results for (NaHal)_15_(Ga_2_S_3_)_23_(GeS_2_)_62_ compositions (Hal = Cl, I) are presented in the Table S1 (Supplementary Materials) and consistent with previous data. In this section we have focused on the characterization of newly synthesized mixed cation AgI-NaI-Ga_2_S_3_-GeS_2_ glasses.

**Figure 1:**
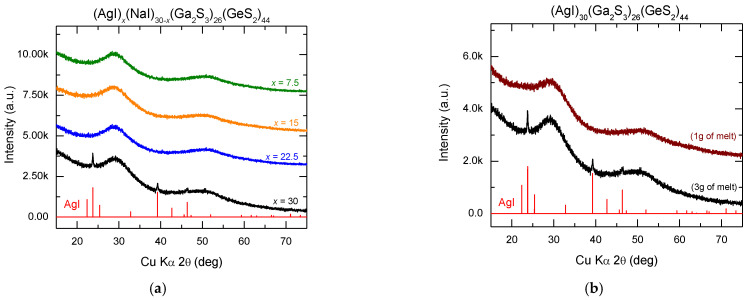
XRD patterns of the (AgI)_x_(NaI)_30−x_(Ga_2_S_3_)_26_(GeS_2_)_44_ glasses: (**a**) x = 7.5, 15, 22.5 and 30 mol.% AgI obtained by quenching 3 g of melt and (**b**) x = 30 mol.% AgI obtained by quenching 1 g and 3 g of melt. The diffraction peaks of hexagonal AgI (JCPDS No. 00-009-0374) are included for comparison.

X-ray diffraction (XRD) patterns of the (AgI)_x_(NaI)_30-x_(Ga_2_S_3_)_26_(GeS_2_)_44_ glasses (where x = 7.5, 15, 22.5 and 30 mol.% AgI) obtained by quenching 3 g of melt are presented in Figure 1a. The diffractions patterns of the sample (AgI)_30_(Ga_2_S_3_)_26_(GeS_2_)_44_ exhibit low intensity Bragg peaks corresponding to crystalline AgI (JCPDS No. 00-009-0374). The X-ray diffraction diagram of the (AgI)_30_(Ga_2_S_3_)_26_(GeS_2_)_44_ glass obtained with a total mass of 1 g exhibit only broad features characteristic of glassy and amorphous materials (Figure 1b). Silver halides doped Ga_2_S_3_-GeS_2_ glasses have been studied previously by several research groups [3,26,27,28,29]. It was reported that the largest amount of dissolved silver iodide can be reached at ≈ 35 mol.% AgI [3,26]. Our results are in accordance with the literature data. The absence of Bragg peaks and the presence of broad diffraction bands confirm the amorphous nature of the (AgI)_x_(NaI)_30−x_(Ga_2_S_3_)_26_(GeS_2_)_44_ glasses where x = 7.5, 15 and 22.5 mol.% AgI (Figure 1a). The amorphous nature of sodium halide doped Ga_2_S_3_-GeS_2_ glasses was already verified in our previous work [13].

The soaking of the (AgI)_x_(NaI)_30−x_(Ga_2_S_3_)_26_(GeS_2_)_44_ glasses in the water over two weeks shows the degradation of the samples with x = 0, 7.5, 15, mol.% AgI. This result is rather unexpected for the (AgI)_15_(NaI)_15_(Ga_2_S_3_)_26_(GeS_2_)_44_ composition, since the single cation sodium containing Ga_2_S_3_-GeS_2_ glasses with 15 mol.% of sodium halide are stable in water [11]. The photos of the samples taken 6 months after soaking are presented in Figure S2 (Supplementary material). We can conclude that the addition of silver cations decreases the moisture resistance of sodium-halide-containing Ga_2_S_3_-GeS_2_ glasses. Thus, the only cation mixed composition stable in water and suitable for the application as sensitive membranes in Na^+^-ISEs is (AgI)_22.5_(NaI)_7.5_(Ga_2_S_3_)_26_(GeS_2_)_44_ glass.

**Figure 2:**
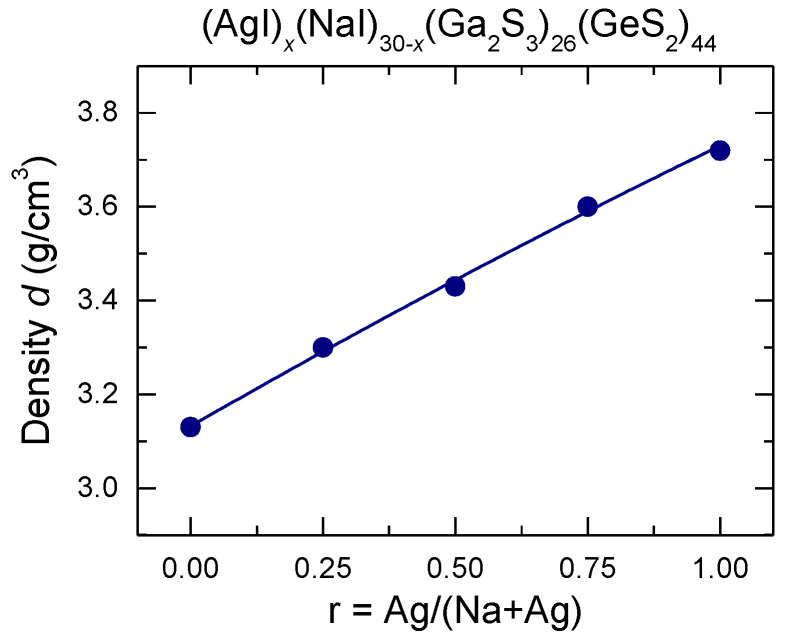
Density of the (AgI)_x_(NaI)_30−x_(Ga_2_S_3_)_26_(GeS_2_)_44_ glasses plotted as a function of the silver fraction r = Ag/(Na + Ag). The solid line is drawn as a guide for the eye.

The measured densities, *d*, of the (AgI)_x_(NaI)_30−x_(Ga_2_S_3_)_26_(GeS_2_)_44_ glasses are listed in Table 1 and plotted in Figure 2 as a function of the silver fraction r = Ag/(Na + Ag). The density increases with increasing silver fraction since the density of silver iodide (*d*_AgI_ = 5.68 g cm^−3^) in higher than that of sodium iodide (*d*_NaI_ = 3.67 g cm^−3^).

**Figure 3:**
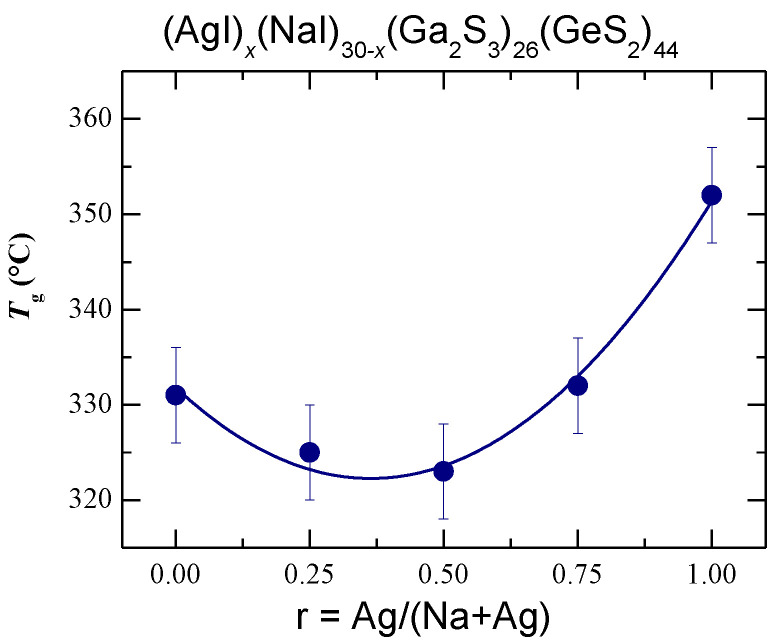
Glass transition temperature, *T*_g_, for the (AgI)_x_(NaI)_30−x_(Ga_2_S_3_)_26_(GeS_2_)_44_ glasses plotted as a function of the silver fraction r = Ag/(Na + Ag). The solid line is drawn as a guide for the eye.

Typical DSC curves of the (AgI)_x_(NaI)_30−x_(Ga_2_S_3_)_26_(GeS_2_)_44_ glasses, x = 0, 7.5, 15, 22.5, and 30 mol.% AgI, are shown in Figure S3 (Supplementary material). All the samples present a single glass transition indicating a homogeneous nature of the synthesized glasses on the macroscopic and mesoscopic scale. The derived glass transition temperatures, *T*_g_, are summarized in Table 1 and plotted in Figure 3 as a function of the silver fraction r = Ag/(Na + Ag). The *T*_g_ changes from 331 °C for (NaI)_30_(Ga_2_S_3_)_26_(GeS_2_)_44_ glass to 352 °C for its silver-containing counterpart and exhibits a slight minimum at *T*_g_ ≈ 323 °C for r ≈ 0.5 or x ≈ 15 mol.% AgI. 

**Figure 4:**
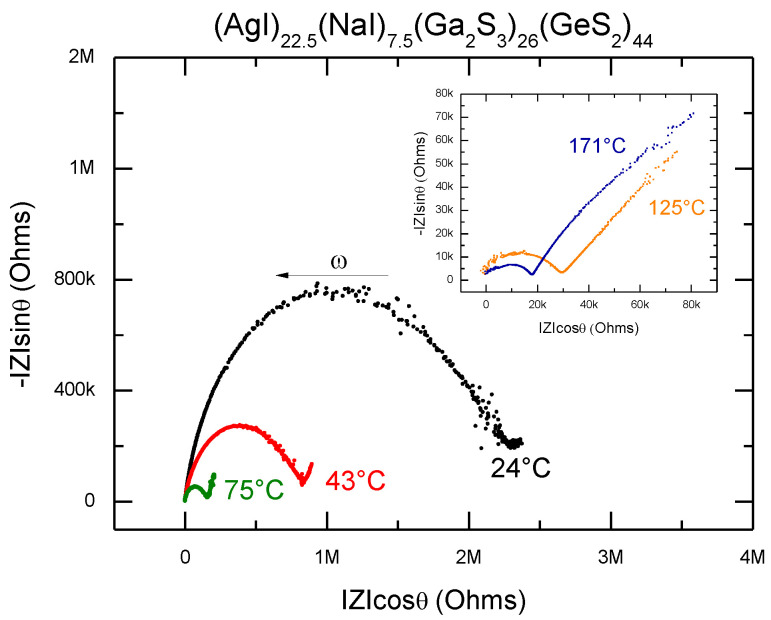
Cole–Cole impedance plots for the (AgI)_22.5_(NaI)_7.5_(Ga_2_S_3_)_26_(GeS_2_)_44_ glass sample at different temperatures.

The total electrical conductivity for all mentioned samples was determined by the complex impedance method. As an example, Figure 4 displays the typical Cole–Cole impedance plots for the (AgI)_22.5_(NaI)_7.5_(Ga_2_S_3_)_26_(GeS_2_)_44_ glass at different temperatures. The sample impedance spectrum consists of a high-frequency distorted semi-circle related to the properties of the sample and a low-frequency polarization tail (increasing with the temperature increase) related to the electrolyte-electrode interface. This behavior is typical for ion-conducting samples in the cell with blocking electrodes. The sample total resistance was determined from the interception of a high-frequency arc with the real axis of the impedance │Z│cosθ. The total electrical conductivity, σ, was obtained by normalizing the sample resistance to a geometrical factor L/S, where L is the sample thickness and S the sample area. 

**Figure 5:**
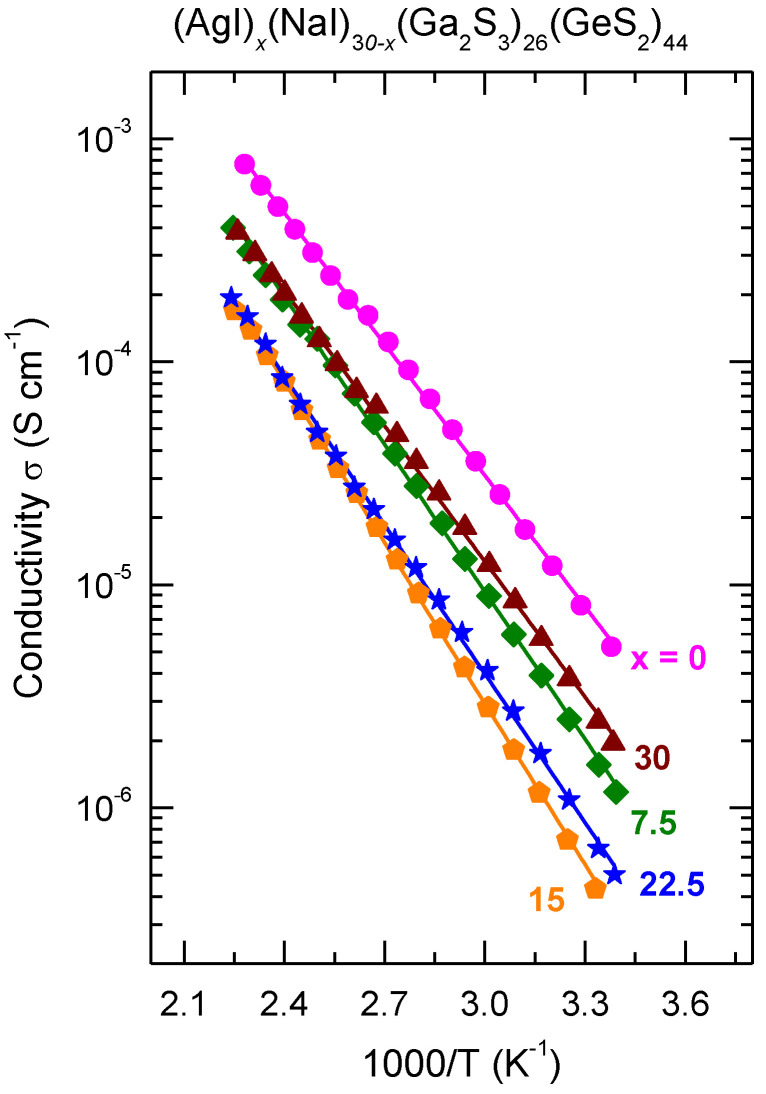
Temperature dependence of the total electrical conductivity σ for the (AgI)_x_(NaI)_30−x_(Ga_2_S_3_)_26_(GeS_2_)_44_ (x = 0; 7.5; 15; 22.5 and 30 mol.% AgI) glasses. The solid lines represent a least-square fit of the data to Equation (2).

**Figure 6:**
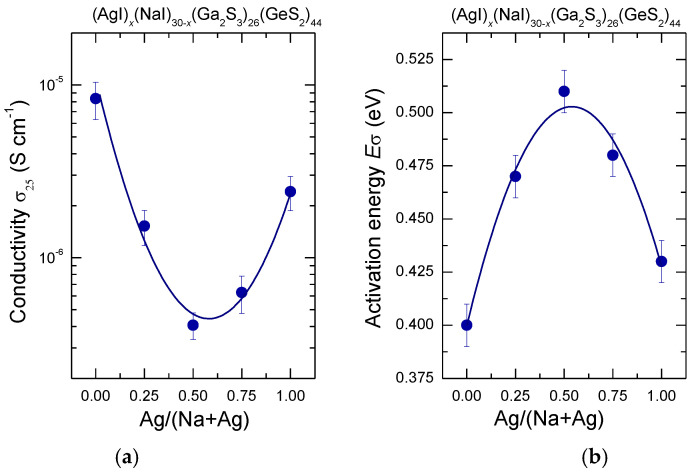
(**a**) Room-temperature conductivity σ_25_ and (**b**) conductivity activation energy *E*_σ_ for the (AgI)_x_(NaI)_30−x_(Ga_2_S_3_)_26_(GeS_2_)_44_ glasses plotted as a function of the silver fraction r = Ag/(Na + Ag). The solid lines are drawn as a guide for the eye.

The conductivity temperature dependences for the (AgI)_x_(NaI)_30−x_(Ga_2_S_3_)_26_(GeS_2_)_44_ (x = 0; 7.5; 15; 22.5 and 30 mol.% AgI) glasses are shown in Figure 5. As usually for this kind of materials, the electrical transport behavior of all the samples obeys the Arrhenius-type equation [30]:

(2) $$\sigma =\frac{A}{T}\exp\left(-\frac{E_{\sigma }}{kT}\right),$$

where *A* is the pre-exponential factor, *E*_σ_ the activation energy, *k* the Boltzmann constant and *T* the temperature. The values of the room-temperature conductivity *σ*_25_, *E*_σ_, and *A* were obtained by linear regression of the data to Equation (2) and listed in Table 1. The results for the room temperature conductivity and the activation energy are plotted as a function of the silver fraction r = Ag/(Na + Ag) in Figure 6. The room-temperature conductivity changes from 8.3 × 10^−6^ S cm^−1^ for (NaI)_30_(Ga_2_S_3_)_26_(GeS_2_)_44_ glass to 2.4 × 10^−6^ S cm^−1^ for its silver relative, passing by a shallow minimum at σ_25_ ≈ 4.1 × 10^−7^ S cm^−1^ for the composition with r ≈ 0.5, Figure 6a. The activation energy changes from 0.4 eV (r = 0) to 0.43 eV (r = 1). The minimum in the conductivity is accompanied by a maximum in the activation energy at *E*_σ_ ≈ 0.51 eV within the same composition range (Figure 6b). The values of the pre-exponential factor, 4.15 ≤ logA ≤ 4.68 (S cm^−1^ K), do not change significantly within the experimental uncertainty (Table 1). The conductivity parameters for single cation MI-Ga_2_S_3_-GeS_2_ (M = Na, Ag) glasses obtained in this work are in accordance with the data reported previously. Indeed, log σ_25_ = −5.00 (2) (S cm^−1^) and *E*_σ_= 0.38 (1) eV were mentioned for (NaI)_30_(Ga_2_S_3_)_26_(GeS_2_)_44_ glass in Ref. [13] and σ_25_ = 1.2 × 10^−6^ S cm^−1^, *E*_σ_= 0.3790(16) eV are given for (AgI)_30_(Ga_2_S_3_)_30_(GeS_2_)_40_ vitreous solid in Ref. [28].

### 3.2. Analytical Characterization of the Sodium Sensors

#### 3.2.1. Slope, Linearity and Detection Limit

**Figure 7:**
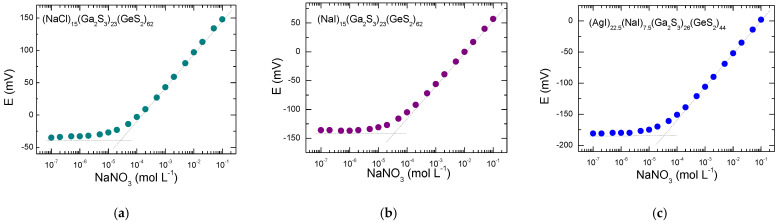
Typical calibration curves for Na^+^ sensors based on (**a**) (NaCl)_15_(Ga_2_S_3_)_23_(GeS_2_)_62_, (**b**) (NaI)_15_(Ga_2_S_3_)_23_(GeS_2_)_62_ and (**c**) (AgI)_22.5_(NaI)_7.5_(Ga_2_S_3_)_26_(GeS_2_)_44_ glass membranes.

The potential response of the fabricated ISEs as a function of the Na*^+^* ions was investigated in the concentration range 10^−7^–10^−1^ M. Figure 7 shows the typical calibration curves for the sensors with (NaCl)_15_(Ga_2_S_3_)_23_(GeS_2_)_62_, (NaI)_15_(Ga_2_S_3_)_23_(GeS_2_)_62_ and (AgI)_22.5_(NaI)_7.5_(Ga_2_S_3_)_26_(GeS_2_)_44_ glass membranes. The lower detection limit was obtained from an intersection of two straight lines of the calibration curve, as shown in Figure 7 by the dashed lines. All the membranes exhibit the comparable analytical characteristics: the detection limits of ~ (2–3) × 10^−5^ M, near-Nernstian linear responses in the concentration range from 10^−4^ to 10^−1^ M sodium nitrate solution, with the slopes of 54–56 mV/decade. However, the calibration of the sensors during a one month period reveals the best stability of the (NaI)_15_(Ga_2_S_3_)_23_(GeS_2_)_62_ based electrodes (Figure S4, Supplementary material). For this reason, the (NaI)_15_(Ga_2_S_3_)_23_(GeS_2_)_62_ membranes were used for further potentiometric characterizations.

#### 3.2.2. Selectivity Coefficient K_Na_^+^_,M_^y+^ in Standard Solutions

The selectivity coefficients of the sensors in the presence of two-valence interfering cations (Mg^2+^, Ca^2+^, Ba^2+^ and Zn^2+^) were determined via a mixed solution method using a constant concentration of the interfering ions and a variable concentration of the Na^+^ ion. In case of one-valence interfering K^+^ ion, the mixed solution method employed a fixed concentration of primary Na^+^ ion and a varying concentration of interfering ions. The second method was chosen to avoid the empoisoning of the sensitive membranes, since the NaCl- Ga_2_S_3_-GeS_2_-based chemical sensors exhibit very low selectivity in the presence of the interfering K^+^ ion [10]. The potentiometric selectivity coefficients (K_Na_^+^_,M_^y+^) of various ions, presented in Table 2 and Table 3, were calculated via the Nikolsky−Eisenman equation [31]:

(3) $$E=E^{0}+\frac{RT}{2F}\;\ln\;({a_{\mathrm{Na}}}^{+}+{K_{\mathrm{Na}}}^{+}{{}_{,\;M}}^{y+}\times a_{M}y^{+})$$

where *R* is the gas constant, *T* the temperature, *F* the Faraday constant, *a*_Na_^+^ the activity of primary ion, *a*_M_^y+^ the activity of interfering M^y+^ ions and y+ charge numbers of various interfering ions. 

Potassium ions interfere strongly with sodium detection, especially in the solutions with a low concentration of Na^+^ ions (Table 3). Other two-valence ions do not affect the Na^+^ ions response appreciably (Table 2). The selectivity coefficients obtained in this work are close to those reported previously for (NaCl)_10_(Ga_2_S_3_)_22.5_(GeS_2_)_67.5_ vitreous membranes [10]. 

#### 3.2.3. PH Influence

**Figure 8:**
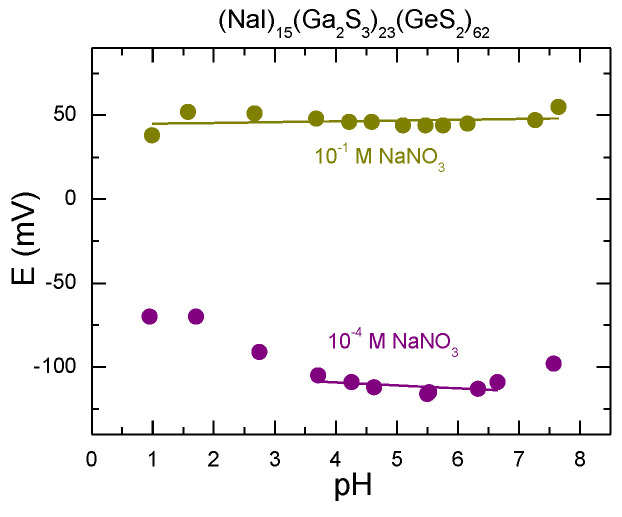
Influence of pH value on the potential of the sensor based on (NaI)_15_(Ga_2_S_3_)_23_(GeS_2_)_62_ glass membrane for two different concentrations of the standard NaNO_3_ solution, 10^−1^ M and 10^−4^ M. The lines are drawn as guides to the eyes.

Figure 8 illustrates the influence of pH on the Na^+^-ISE based on (NaI)_15_(Ga_2_S_3_)_23_(GeS_2_)_62_ glass membrane. The measurements were performed for two different concentrations of Na^+^ primary ions, 1 × 10^−1^ M and 1 × 10^−4^ M NaNO_3_, in the pH range between 1 and 8. For the diluted sodium nitrate concentration (1 × 10^−4^ M NaNO_3_), the potential is constant in the pH range between 3.5 and 7. The working pH range improves when the concentration of the NaNO_3_ solution is increased (1 × 10^−1^ M), and the potential is nearly constant over the whole measured pH range from 1 to 8.

#### 3.2.4. Reproducibility of the Electrode Potential

**Figure 9:**
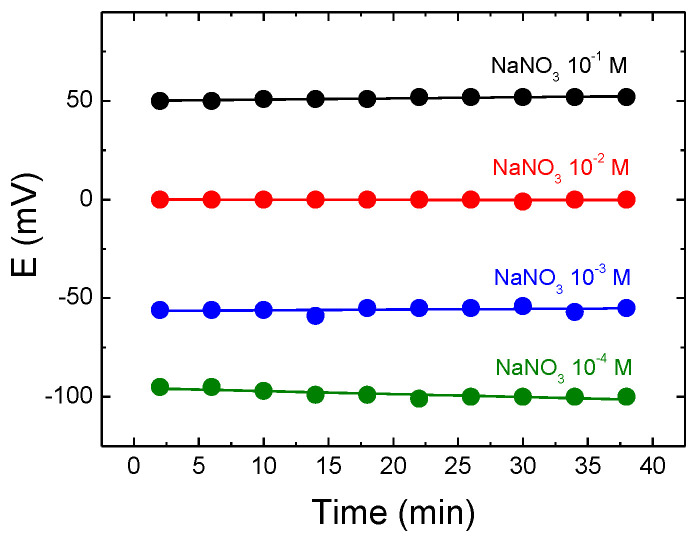
The reproducibility of the sensor based on (NaI)_15_(Ga_2_S_3_)_23_(GeS_2_)_62_ glass membrane in different concentrations of the standard NaNO_3_ solution. The lines are drawn as guides to the eyes.

The reproducibility parameter, primordial for continuous in situ measurements, was investigated using a series of consecutive measurements in standard solutions [32]. Figure 9 shows the reproducibility of the sensor based on the (NaI)_15_(Ga_2_S_3_)_23_(GeS_2_)_62_ glass membrane for four different concentrations of the NaNO_3_ solution, from 10^−4^ M to 10^−1^ M. The sensor measures the potential of the solution of a given concentration for a period 2 min and is then placed in the open air for another 2 min. This procedure is repeated ten times in order to study the reproducibility of the potential as a function of time. Figure 9 shows that the change in potential, during consecutive measurements, is ± 2–3 mV and that the reproducibility does not depend on the concentration of the primary Na^+^ ions.

## 4. Discussion

### 4.1. Mixed Cation Effect Phenomenon in the (AgI)_x_(NaI)_30−x_(Ga_2_S_3_)_26_(GeS_2_)_44_ Glasses

In general, mixed cation effect corresponds to a non-monotonic variation of the glass properties associated with the ion motion (such as ionic conductivity and its activation energy, cation diffusion, or viscosity) when one cation is successively replaced by another at a fixed total cation content [33,34,35]. The purpose of this research was to confirm the presence of the mixed cation effect in the mixed silver/sodium iodide doped Ga_2_S_3_-GeS_2_ glasses by the measurements of electrical conductivity and glass transition temperature. The obtained results of macroscopic and electrical properties in the (AgI)*_x_*(NaI)_30-*x*_(Ga_2_S_3_)_26_(GeS_2_)_44_ system indicated mixed cation effect with the minimum of both glass transition temperature (Figure 3) and room temperature conductivity (Figure 6a), and the activation energy maximum (Figure 6b), centered at *r* = Ag/(Na + Ag) ≈ 0.5. As expected, the density is not affected by cation mixing and increases monotonically between two end-members when NaI is progressively replaced by AgI (Figure 2). 

According to the previous research works, the depth and the position of the minimum in *T*_g_ and *σ* of the mixed cation glasses depend on the nature of mobile ions. The light alkali mixed *x*Na_2_S-(0.5-*x*)Li_2_S-0.5SiS_2_ [16] and *x*Na_2_S-(0.5-*x*)Li_2_S-0.5GeS_2_ [18] glasses show a deep conductivity minimum of 2.5 orders of magnitude at equimolar cation ratio Na/(Li + Na) ≈ 0.5, while the heavy cations mixed glasses (for example Ag^+^/Rb^+^ [20] and Ag^+^/Tl^+^ [22,23] thiogermanates with a significant difference in ion size, and therefore in ionic mobility between silver and rubidium or thallium cations) exhibit a shallow conductivity minimum at room temperature shifted to Ag-poor compositions. The similar situation was observed for *x*Na_2_S-(0.56-*x*)Rb_2_S-0.44SiS_2_ glasses, where the minimum of approximately two orders of magnitude in *σ* at 149.6 °C occurs at a concentration ratio Na/(Rb + Na) of about 0.4 to 0.45 [15]. It should be also noted that the magnitude of the conductivity minimum is temperature dependent and decreases with the temperature increase [15,36]. In this work, the conductivity of silver iodide doped Ga_2_S_3_-GeS_2_ glasses is comparable to that of sodium-iodide-containing counterparts. Both silver and sodium cations possess rather high ionic mobility due to their small ion size. Thus, our results show a manifestation of the mixed cation effect centered at r ≈ 0.5, similar to Na^+^/Li^+^ mixed glasses, with a relatively shallow conductivity minimum of one order of magnitude. This shallow minimum could be explained by rather low total cation content in the (MI)_30*−x*_(Ga_2_S_3_)_26_(GeS_2_)_44_ (M = Ag, Na) glasses with ≈9.32 at.% M. 

The addition of silver iodide to NaI containing Ga_2_S_3_-GeS_2_ glasses leads to the degradation of their moisture resistance (Figure S2, Supplementary material). Indeed, the single cation sodium containing Ga_2_S_3_-GeS_2_ glasses with 15 mol.% of sodium halide are stable in aqueous solutions and were used in this work for chemical sensors applications, while the samples with both 15 mol.% of NaI and 15 mol.% of AgI crumbled in several days of soaking in water. This fact is unexpected, since silver-containing chalcogenide glasses are known for their enhanced atmospheric stability.

### 4.2. The Performance of the Sodium Sensors with Different Membrane Compositions

Among three different membrane compositions studied in this work, i.e., (NaCl)_15_(Ga_2_S_3_)_23_(GeS_2_)_62_, (NaI)_15_(Ga_2_S_3_)_23_(GeS_2_)_62_ and (AgI)_22.5_(NaI)_7.5_(Ga_2_S_3_)_26_(GeS_2_)_44_ glasses, the (NaI)_15_(Ga_2_S_3_)_23_(GeS_2_)_62_ vitreous alloy seems to be more suitable for Na^+^-ISE applications due to its better aging stability (Figure S4, Supplementary material). However, the detection limit (~(2–3) × 10^−5^ M) and the sensitivity (54–56 mV decade^−1^) are similar for all studied materials. The sensor performance is slightly enhanced compared to previous results for (NaCl)_10_(Ga_2_S_3_)_22.5_(GeS_2_)_67.5_ and (NaY)_15_(Ga_2_S_3_)_17_(GeS_2_)_68_ (Y = Cl, Br, I) based sensors [10,11]. 

The composition of the glassy matrix seems to affect Na^+^ ion response. Thus, the increase in the Ga_2_S_3_/GeS_2_ ratio improves the sodium detection. The monotonic decrease in sodium chloride content and an enhancement of the Ga/Ge ratio on the glass surface after one day soaking, revealed by X-ray photoelectron spectroscopy investigation of the (NaCl)*_x_*(Ga_2_S_3_)_25−0.25*x*_(GeS_2_)_75−0.75*x*_ (*x* = 10, 20) vitreous alloys [10], confirm the chemical stability of gallium rich glassy matrix. 

The variation of the halide nature and the sodium halide content could affect the analytical characteristics of the sensors to some extent. The ^22^Na tracer measurements showed that the mechanism of ionic sensitivity of chalcogenide glass electrodes is based on the Na^+^ ion-exchange between solution and glass membrane, and the sodium diffusion in the surface layer of the glass is the rate-determining step of Na^+^ ion-exchange [10]. It is generally accepted that the halide nature affect the conductivity of the alkali halide doped glasses weakly [13]. Nevertheless, the increase in conductivity with increasing halogen ion size was reported for AgBr–As_2_Se_3_ and AgI–As_2_Se_3_ chalcogenide glassy systems [37,38]. Taking into account these data, we could suppose that the sodium iodide doped glasses are more promising for sensors applications. The ionic conductivity increases with sodium content, and a distinct saturation is observed for the concentrations above 20 mol.% NaY [13]. Nevertheless, the appropriate sodium halide content in glassy membranes is 15 mol.%, since the glasses with the concentrations above 15 mol.% NaY are not stable in aqueous solutions.

Finally, the variation of the halide nature, Ga_2_S_3_/GeS_2_ ratio, and the sodium content in the sodium halide doped Ga_2_S_3_-GeS_2_ vitreous membranes could improve the performance of the sodium sensors slightly. The glasses with 15 mol.% NaI and Ga_2_S_3_/GeS_2_ ratio ≈ 0.37 seem to be more suitable for sensors applications. In comparison with previous studies [10,11], the detection limit for the ion-selective electrodes with the glasses presented in this work could be nearly an order of magnitude lower.

## 5. Conclusions

(AgI)*_x_*(NaI)_30−*x*_(Ga_2_S_3_)_26_(GeS_2_)_44_ chalcogenide glasses (where *x* = 0, 7.5, 15, 22.5 and 30 mol.% AgI) were synthesized and characterized using density, DSC, and conductivity measurements. The mixed cation effect with shallow conductivity and glass transition temperature minimum was found for silver fraction r = Ag/(Na + Ag) ≈ 0.5. Silver addition decreases the moisture resistance of the glasses. Only (AgI)_22.5_(NaI)_7.5_(Ga_2_S_3_)_26_(GeS_2_)_44_ composition was suitable for chemical sensors application, contrary to the single cation sodium halide doped Ga_2_S_3_-GeS_2_ glasses, where 15mol.% sodium-halide-containing vitreous alloys are stable in water solutions. The analytical parameters of (NaCl)_15_(Ga_2_S_3_)_23_(GeS_2_)_62_, (NaI)_15_(Ga_2_S_3_)_23_(GeS_2_)_62_ and (AgI)_22.5_(NaI)_7.5_(Ga_2_S_3_)_26_(GeS_2_)_44_ glasses as active membranes in Na^+^-ISEs were investigated. The detection limit (~(2–3) × 10^−5^ M) and the sensitivity (54–56 mV decade^−1^) are similar for all studied compositions. However, the (NaI)_15_(Ga_2_S_3_)_23_(GeS_2_)_62_ vitreous alloy seems to be more suitable for Na^+^-ISE applications due to its better stability after one month calibration. This sensor shows an excellent reproducibility, good pH working range, and good selectivity and reversibility to Na^+^ ions in the presence of Mg^2+^, Ca^2+^, Ba^2+^ and Zn^2+^ interfering cations. However, the (NaI)_15_(Ga_2_S_3_)_23_(GeS_2_)_62_-based sensor was sensitive to K^+^ interfering ions. The variation of the halide nature, the sodium halide content and the glassy matrix composition could affect the sensor’s response.

## Figures and Tables

**Table 1:** Macroscopic properties of the (AgI)*_x_*(NaI)_30−*x*_(Ga_2_S_3_)_26_(GeS_2_)_44_ glasses: density, *d*, glass transition temperature, *T*_g_, the room-temperature conductivity *σ*_25_, the activation energy *E_σ_*, and the pre-exponential factor *A* ^1^.

Glass Composition		*d* (g cm^−3^)	*T*_g_ (°C)	log *σ*_25_ (S cm^−1^)	*E_σ_* (eV)	log *A* (S cm^−1^ K)
*x*, mol.% AgI	r = Ag/(Na + Ag)					
0	0	3.13 (2)	331 (5)	−5.08 (2)	0.40 (1)	4.15 (4)
7.5	0.25	3.30 (2)	325 (5)	−5.82 (1)	0.47 (1)	4.58 (4)
15	0.5	3.43 (2)	323 (5)	−6.39 (1)	0.51 (1)	4.68 (3)
22.5	0.75	3.60 (2)	332 (5)	−6.20 (3)	0.48 (1)	4.46 (8)
30	1	3.72 (2)	352 (5)	−5.62 (1)	0.43 (1)	4.17 (4)

^1^ Uncertainties in the last digit(s) of the parameter are given in parentheses.

**Table 2:** Selectivity coefficients of Na^+^-ISE based on (NaI)_15_(Ga_2_S_3_)_23_(GeS_2_)_62_ glass membrane with respect to the interfering ions Mg^2+^, Ca^2+^, Ba^2+^ and Zn^2+^. The measurements were conducted using a constant concentration of interfering ions ^1^.

Interfering Ion	Interfering Ion Concentration (mol L^−1^)	Selectivity Coefficient K_Na_^+^_,M_^2+^
Mg^2+^	1	2.3 (5) × 10^−4^
Ca^2+^	1	1.4 (7) × 10^−4^
Ba^2+^	0.1	5.8 (4) × 10^−4^
Zn^2+^	1	4.7 (4) × 10^−4^

^1^ Uncertainties in the last digit of the parameter are given in parentheses.

**Table 3:** Selectivity coefficients of Na^+^-ISE based on (NaI)_15_(Ga_2_S_3_)_23_(GeS_2_)_62_ glass membrane with respect to the interfering ions K^+^. The measurements were conducted using a constant concentration of Na^+^ ions ^1^.

Na^+^ Primary Ion Concentration (mol L^−1^)	Selectivity Coefficient K_Na_^+^_,K_^+^
1 × 10^−4^	1.5 (2) × 10^−1^
1 × 10^−3^	1.9 (2) × 10^−1^
1 × 10^−2^	5.6 (3) × 10^−2^
1 × 10^−1^	1.7 (3) × 10^−2^

^1^ Uncertainties in the last digit of the parameter are given in parentheses.

## Data Availability

Not applicable.

## Supplementary Materials

The following supporting information can be downloaded at: https://www.mdpi.com/article/10.3390/s22249986/s1, Figure S1: The photos of the typical AgI-NaI-Ga_2_S_3_-GeS_2_ glasses and Na^+^-ISE taking the (AgI)_22.5_(NaI)_7.5_(Ga_2_S_3_)_26_(GeS_2_)_44_ sample as an example: (a) the silica tube (ID/OD of 8/10 mm) quenched horizontally with 3g of the melt; (b) the semi-spherical shaped membrane for ISE obtained by vertical quenching of the silica tube (ID/OD of 10/12 mm) with 1.5 g of the sample; (c) the fabricated liquid contact chemical sensor with the sensitive membrane glued on the PVC tube filled with the NaCl (0.1 M) solution, and the AgCl coated silver wire; Figure S2: The photos of the (AgI)*_x_*(NaI)_30-*x*_(Ga_2_S_3_)_26_(GeS_2_)_44_ samples, *x* = 0, 7.5, 15, 22.5 and 30 mol.% AgI, taken 6 months after soaking in water for 2 weeks; Figure S3: Typical DSC curves of the (AgI)*_x_*(NaI)_30−*x*_(Ga_2_S_3_)_26_(GeS_2_)_44_ glasses, *x* = 0, 7.5, 15, 22.5 and 30 mol.% AgI; Figure S4: Calibration curves obtained during one month period for Na^+^ sensors based on (a) (NaCl)_15_(Ga_2_S_3_)_23_(GeS_2_)_62_, (b) (NaI)_15_(Ga_2_S_3_)_23_(GeS_2_)_62_ and (c) (AgI)_22.5_(NaI)_7.5_(Ga_2_S_3_)_26_(GeS_2_)_44_ glass membranes; Table S1: Macroscopic properties of the (NaHal)_15_(Ga_2_S_3_)_23_(GeS_2_)_62_ (Hal = Cl, I) compositions: density, *d*, glass transition temperature, *T*_g_, the room-temperature conductivity *σ*_298_, the activation energy *E_σ_*, and the pre-exponential factor *A.*
