# Supplementary material for: Potentiometric Chemical Sensors Based on Metal Halide Doped Chalcogenide Glasses for Sodium Detection
Source: Sensors (Basel). 2022 Dec 18;22(24):9986. doi: 10.3390/s22249986 (PMC9785170; doi:10.3390/s22249986)
Supplement: Supplementary file 1 [file sensors-22-09986-s001.zip › sensors-2082992-supplementary.pdf]

Supplementary Materials

Potentiometric chemical sensors based on metal halide doped chalcogenide glasses for sodium detection

Maria Bokova [1,*], Steven Dumortier [1], Christophe Poupin[2], Renaud Cousin[2], Mohammad Kassem [1] and Eugene Bychkov [1]

[1] Université du Littoral Côte d'Opale (ULCO), Laboratoire de Phisico-Chimie de l'Atmosphère (LPCA), EA 4493, 59140 Dunkerque, France

[2] Université du Littoral Côte d'Opale (ULCO), Unité de Chimie Environnementale et Interactions sur le Vivant (UCEIV), UR 4492, SFR Condorcet FR CNRS 3417, 59140 Dunkerque, France

* Correspondence: Maria.Bokova@univ-littoral.fr; Tel.: +33 2 28 65 82 70

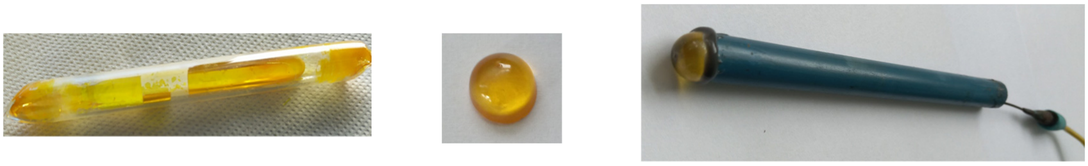

(a) (b) (c)

Figure S1. The photos of the typical AgI-NaI-Ga_2S_3-GeS_2 glasses and Na^+-ISE taking the $(AgI)_{22.5}(NaI)_{7.5}(Ga_2S_3)_{26}(GeS_2)_{44}$ sample as an example: (a) the silica tube (ID/OD of 8/10 mm) quenched horizontally with 3g of the melt; (b) the semi-spherical shaped membrane for ISE obtained by vertical quenching of the silica tube (ID/OD of 10/12 mm) with 1.5 g of the sample; (c) the fabricated liquid contact chemical sensor with the sensitive membrane glued on the PVC tube filled with the NaCl (0.1 M) solution and the AgCl coated silver wire.

Table S1. Macroscopic properties of the $(NaHal)_{15}(Ga_2S_3)_{23}(GeS_2)_{62}$ (Hal = Cl, I) compositions: density, *d*, glass transition temperature, T_g, the room-temperature conductivity σ_{298}, the activation energy E_σ, and the pre-exponential factor *A*[1].

Glass composition	*d* (g cm^{-3})	T_g (°C)	log σ_{298} (S cm^{-1})	E_σ (eV)	log *A* (S cm^{-1} K)
Y = Cl	2.86(2)	355(10)	-7.13(6)	0.48(2)	3.48(17)
Y = I	3.03(2)	364(10)	-6.18(2)	0.44(1)	3.80(6)

[1] Uncertainties in the last digit(s) of the parameter are given in parentheses.

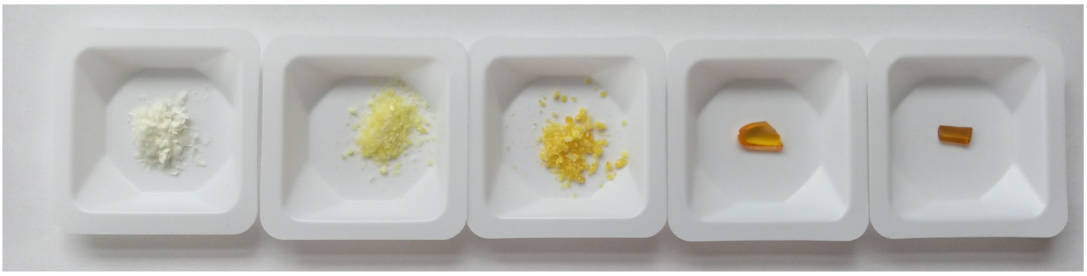

$x = 0$ $x = 7.5$ $x = 15$ $x = 22.5$ $x = 30$

Figure S2. The photos of the $(AgI)_x(NaI)_{30-x}(Ga_2S_3)_{26}(GeS_2)_{44}$ samples, x = 0, 7.5, 15, 22.5 and 30 mol.% AgI, taken 6 months after soaking in water during 2 weeks.

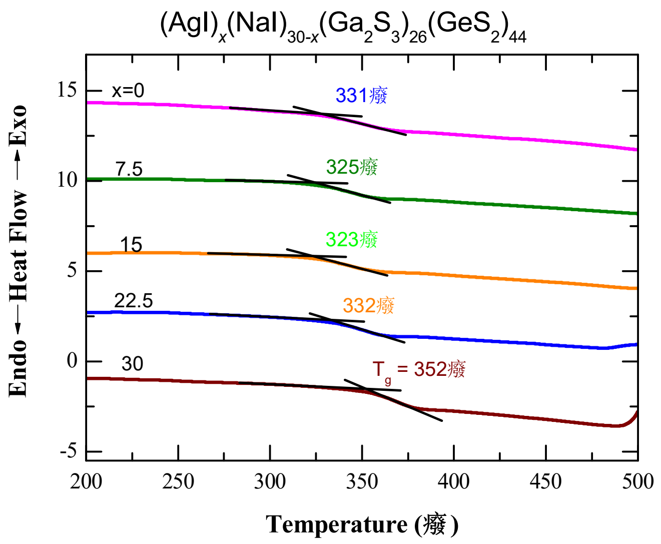

Figure S3. Typical DSC curves of the $(AgI)_x(NaI)_{30-x}(Ga_2S_3)_{26}(GeS_2)_{44}$ glasses, x = 0, 7.5, 15, 22.5 and 30 mol.% AgI.

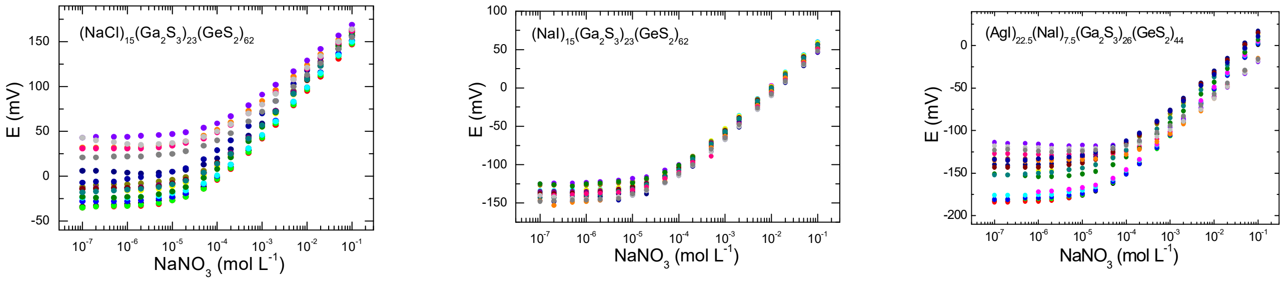

(a) (b) (c)

Figure S4. Calibration curves obtained during one month period for Na^+ sensors based on (a) $(NaCl)_{15}(Ga_2S_3)_{23}(GeS_2)_{62}$, (b) $(NaI)_{15}(Ga_2S_3)_{23}(GeS_2)_{62}$ and (c) $(AgI)_{22.5}(NaI)_{7.5}(Ga_2S_3)_{26}(GeS_2)_{44}$ glass membranes.
